# A Novel Approach to Design and Evaluate BNCT Neutron Beams Combining Physical, Radiobiological, and Dosimetric Figures of Merit

**DOI:** 10.3390/biology10030174

**Published:** 2021-02-26

**Authors:** Ian Postuma, Sara González, Maria S Herrera, Lucas Provenzano, Michele Ferrarini, Chiara Magni, Nicoletta Protti, Setareh Fatemi, Valerio Vercesi, Giuseppe Battistoni, Umberto Anselmi Tamburini, Yuan Hao Liu, Leena Kankaanranta, Hanna Koivunoro, Saverio Altieri, Silva Bortolussi

**Affiliations:** 1 Istituto Nazionale di Fisica Nucleare (INFN), Unit of Pavia, via Bassi 6, 27100 Pavia, Italy; chiara.r.magni@gmail.com (C.M.); Nicoletta.protti@pv.infn.it (N.P.); fatemi.setareh@gmail.com (S.F.); Valerio.vercesi@pv.infn.it (V.V.); tau@unipv.it (U.A.T.); Saverio.altieri@pv.infn.it (S.A.); silva.bortolussi@pv.infn.it (S.B.); 2 Comisión Nacional de Energía Atómica (CNEA), Avenida General Paz 1499, Villa Maipú, Buenos Aires B1650, Argentina; srgonzal@cnea.gov.ar (S.G.); lucasprovenzano@hotmail.com (L.P.); 3 Consejo Nacional de Investigaciones Científicas y Técnicas (CONICET), Godoy Cruz 2290, Argentina; mariettaherrera@gmail.com; 4 Centro Nazionale di Adroterapia Oncologica, CNAO, Strada Campeggi 53, 27100 Pavia, Italy; michele.ferrarini@cnao.it; 5 Department of Physics, University of Pavia, via Bassi 6, 27100 Pavia, Italy; 6 Istituto Nazionale di Fisica Nucleare (INFN), Unit of Milan, via Celoria 16, 20133 Milan, Italy; giuseppe.battistoni@mi.infn.it; 7 Department of Chemistry, University of Pavia, via Taramelli 12, 27100 Pavia, Italy; 8 Neuboron Medtech, No. 568, Longmian Ave., Jiangning District, Nanjing 210093, China; yhl.taiwan@gmail.com; 9 Department of Nuclear Science and Technology, Nanjing University of Areonautics and Astronautics, Nanjing 210093, China; 10 Department of Oncology, Helsinki University Hospital and University of Helsinki, Yliopistonkatu 4, 00100 Helsinki, Finland; Leena.kankaanranta@hus.fi; 11 Neutron Therapeutics, 1 Industrial Drive, Danvers, MA 01923, USA; hanna.koivunoro@helsinki.fi

**Keywords:** BNCT, BSA, UTCP, epithermal neutron beam, radiobiological figure of merit, out-of-beam dosimetry

## Abstract

**Simple Summary:**

Clinical potential and safety are presented as novel criteria to evaluate neutron beams designed for boron neutron capture therapy (BNCT). The presently used figures of merit are a set of physical quantities calculated in air, related to the neutron flux, the collimation, and the spectral characteristics. However, the capability of the beam to deliver an effective and safe treatment to patients should be the most important criterion in view of the clinical application. This work presents the design of a neutron beam produced by a proton accelerator coupled to a beryllium target and the use of new figures of merit to choose the best beam among different candidates. These figures of merit use tridimensional dosimetry simulated in phantoms and patients, to calculate the probability of tumor control without affecting healthy tissues, employing proper radiobiological models. Moreover, the dose absorbed by out-of-field healthy organs is used as a criterion to establish the safest beam for clinical treatments. Results show that beams that would be rejected by physical in-air quantities demonstrate a clinical performance comparable to existing neutron beams successfully used for patients, and that the presented criteria allow a clear selection of the most adequate beam among the ones presented.

**Abstract:**

(1) Background:The quality of neutron beams for Boron Neutron Capture Therapy (BNCT) is currently defined by its physical characteristics in air. Recommendations exist to define whether a designed beam is useful for clinical treatment. This work presents a new way to evaluate neutron beams based on their clinical performance and on their safety, employing radiobiological quantities. (2) Methods: The case study is a neutron beam for deep-seated tumors from a 5 MeV proton beam coupled to a beryllium target. Physical Figures of Merit were used to design five beams; however, they did not allow a clear ranking of their quality in terms of therapeutic potential. The latter was then evaluated based on in-phantom dose distributions and on the calculation of the Uncomplicated Tumor Control Probability (UTCP). The safety of the beams was also evaluated calculating the in-patient out-of-beam dosimetry. (3) Results: All the beams ensured a UTCP comparable to the one of a clinical beam in phantom; the safety criterion allowed to choose the best candidate. When this was tested in the treatment planning of a real patient treated in Finland, the UTCP was still comparable to the one of the clinical beam. (4) Conclusions: Even when standard physical recommendations are not met, radiobiological and dosimetric criteria demonstrate to be a valid tool to select an effective and safe beam for patient treatment.

## 1. Introduction

Boron Neutron Capture Therapy (BNCT) is a form of hadrontherapy that exploits the products of neutron capture reactions in ${}^{10}$B to treat neoplasms. The therapy is performed by targeting the tumor with a drug loaded with ${}^{10}$B and subsequently irradiating the volume with a thermal neutron field (i.e., 25 meV neutrons). At this energy, the thermal neutron capture reaction ${}^{10}B{\left(n,\alpha \right)}^{7}Li$ has a high cross Section (3837 b) and generates two high-LET, short-range charged particles, whose damage is localized in the proximity of cells that uptake ${}^{10}$B. If boron concentration is sufficiently higher in the malignancy than in the normal tissues, it is possible to cause a selective lethal effect in tumors. The selectivity warranted by the biological distribution of boron makes BNCT a potential therapeutic option for disseminated or infiltrated neoplasms. Moreover, as the effect is due to highly biological effective radiation, BNCT is studied to treat recurrent or radio-resistant tumors. Many BNCT clinical trials have been performed worldwide with promising results, especially on head and neck cancer, cutaneous melanoma, and brain tumors [1,2,3].

BNCT has been applied in institutions equipped with research nuclear reactors. Through appropriate Beam Shaping Assemblies (BSA), thermal or epithermal neutron beams with fluxes of the order of 10${}^{9}$ cm${}^{-2}$ s${}^{-1}$ can be delivered on the patient. These beams are intense enough to ensure acceptable treatment times respectively for shallow and deep seated tumors. Today, a neutron flux of the same order can be produced with proton accelerators (accelerator-based BNCT or ab-BNCT) coupled with Be or Li targets followed by a BSA tailored to obtain the desired neutron spectra. The BSA is always necessary unless neutrons are fully thermalized before irradiating the patient [4,5]. Accelerators are more compact than reactors, easier to operate and maintain, and have no social acceptability issues, allowing their installation in health-care environments. This technology is giving a worldwide boost to new BNCT clinical facilities. Cyclotrons for BNCT are already in use for clinical trials in two centers in Japan [6]. Other accelerators, electrostatic or Radio Frequency Quadrupole (RFQ), are being developed and installed in Argentina, Japan, Russia, Finland, USA, UK, and Israel [7,8,9,10,11,12,13].

The Italian National Institute of Nuclear Physics (INFN) designed and constructed an RFQ proton accelerator delivering a 5 MeV, 30 mA proton beam in Continuous Wave (CW) mode [14]. The neutron yield at the Be target is 10${}^{14}$ s${}^{-1}$ with maximum neutron energy around 3.2 MeV [15]. This spectrum must be tailored for BNCT, acting on moderation and collimation.

The optimal neutron beam for BNCT of deep-seated tumors has a spectrum peaked between 1 and 10 keV [16,17,18], requiring a BSA to decrease neutron energy and to collimate the beam to minimize out-of-beam dose. The BSA is a typical element of the BNCT equipment, both for accelerators and reactors, and its characteristics depend on the initial neutron energy distribution (i.e., on the type of target and on the projectile energy) in the accelerator case. Presently, there is an increasing research work designing BSAs for ab-BNCT [13,15,19,20,21,22,23,24,25,26]. Hence, methods to compare and evaluate simulated beams are currently under discussion, introducing new simulation instruments, computational strategies, and analysis criteria.

IAEA published the TecDoc-1223 in 2001 [27] describing the desired characteristics of the neutron beams for BNCT, values had been indicated for:

- minimum epithermal neutron flux, $\phi_{epi}$ > 10${}^{9}$ (cm${}^{-2}$ s${}^{-1}$).
- minimum degree of collimation as neutron current over epithermal neutron flux $\frac{J}{\phi_{epi}}$ > 0.7
- maximum gamma dose rate epithermal neutron flux $\frac{{\dot{D}}_{\gamma }}{\phi_{epi}}$ < 2 · 10${}^{-13}$ (cm${}^{2}$ Gy)
- maximum thermal neutron and epithermal neutron flux ratio $\frac{\phi_{th}}{\phi_{epi}}$ < 0.05
- maximum fast neutron dose rate over epithermal neutron flux $\frac{{\dot{D}}_{Fast}}{\phi_{epi}}$ < 2 · 10${}^{-13}$ (cm${}^{2}$ Gy)

Together with some in-phantom quantities [28], these Figures of Merit (FOM) until now have been the only guidelines to evaluate the suitability of neutron beams for BNCT. Presently, the idea is taking shape that a neutron beam should rather be evaluated using treatment planning calculations in real clinical cases for a reliable performance evaluation: beams that do not comply with the IAEA guidelines can still be effective for some tumors. Furthermore, a neutron beam for BNCT must also follow other important criteria: it must be safe for the whole body of the patient, and it must be tested for the radio-protection limitations according to the regulations in each country. In literature, few examples of more refined computational evaluations are available [29,30].

In this work, beams obtained with different BSA configurations have been first evaluated according to the classic IAEA FOMs. For a deeper analysis, we then exploited a radiobiological figure of merit as an indicator of their clinical performance. The same beams were then tested on a human whole-body phantom to evaluate the dose delivered to peripheral organs. The information retrieved from these evaluations led to the selection of the best BSA configuration. The candidate beam was finally used to simulate a treatment plan in a patient bearing head and neck tumors, one of the most promising targets in present BNCT clinical trials. The result of the radiobiological figure of merit in the treatment planning was compared with that of the original BNCT treatment performed at FiR1 in Finland.

The goal of these evaluations is to provide a more realistic clinical assessment of the designed neutron beams, allowing the selection of the optimal candidate in terms of therapeutic potential and safety.

## 2. Materials and Methods

Presently many reactor and accelerator-based neutron sources are available, all with characteristic neutron spectra, needing ad hoc BSA materials and geometrical configuration to make them suitable for BNCT. The INFN RFQ accelerator produces neutrons by interactions of 5 MeV protons with a Be target. The target is described in [15], and it has been fully modeled in the simulation, comprising the cooling system. The neutron source has been simulated and validated using double-differential spectra measured for the Be(p,n) reaction at 5 MeV [31,32]. Given the produced spectrum, the most performing moderating material has been proved to be aluminum fluoride (AlF${}_{3}$) [32], which is available only in powder. To obtain a mechanically stable BSA, a dedicated study was carried out in Pavia to densify powders of AlF${}_{3}$, added with LiF. An innovative sintering process has been devised and a dedicated machine has been designed and constructed, able to obtain, for the first time, aluminum fluoride elements with nearly 100% density, showing very good mechanical properties and resistance to radiation. AlF${}_{3}$ doped with LiF is used throughout this work as the bulk moderation unit, to control the epithermal neutron flux and to lower as much as possible the fast (>10 keV) and thermal (<0.5 eV) neutron components. For simplicity, we do not present all the tested BSA materials and geometrical configurations. Instead, only five set-ups are reported to show the approach used to select the most performing BSA.

### 2.1. BSA Material and Geometrical Composition

**Figure 1:**
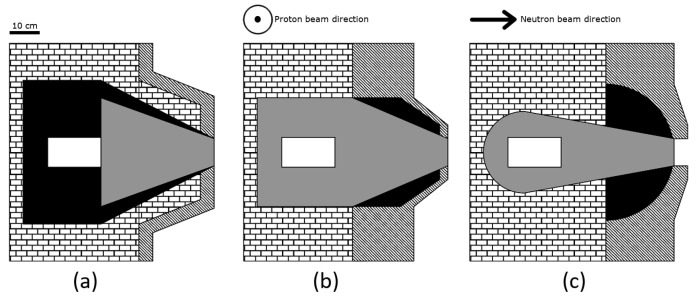
Schemes of the tested BSA structures. Each BSA can be separated into four different regions: The brick pattern is a first shielding material (shield 1), the black region is the reflector, the striped area represents a second shielding material (shield 2), and the grey area is the bulk moderating material. The central white box is the position of the target, neutrons are moderated and transported horizontally from the white box towards the beam port on the right. These schemes are a focus in the main part of the BSA. Lateral dimensions (i.e., the cube containing the bulk material) are 160 cm by 160 cm; for the horizontal extension, refer to Table 1. The 3D structure has a cylindrical symmetry and can be reproduced by rotating the images horizontally around their central axis. The neutron source is located inside the white rectangle at the center of the BSA, and the proton beam is perpendicular to the direction of the neutron beam. In set-up (**a**) the bulk moderating material is enclosed in the reflector material which is in turn surrounded by shield 1 and 2. This configurations has the most pronounced cone shape and least shielding. Set-up (**b**) has the bulk moderating material surrounding the neutron source which is then enveloped by shielding 1 and 2. The reflector is limited to the cone region. This configurations ensures an higher lateral shielding with respect to the previous BSA with a little loss of neutron flux at the beam port. Finally, configuration (**c**) is similar to (**b**) but with an increased reflector and shielding 2 region. This decreases the cone shape of the BSA and drastically decreases unwanted radiation escaping from the BSA, this also leads to much less neutrons at the beam port.

Figure 1 shows three geometrical configurations, selected based on previous experience regarding the necessity to reflect neutrons to increase the flux, to absorb unwanted spectral components and to collimate the beam. The set-up in (a) has a reflector (black) that embeds the bulk moderating material (grey) which is embedded into shield 1 (bricks), aimed at decreasing lateral neutron and gamma contamination. In (b), the bulk material embeds the target to guide neutrons with the desired energy towards the beam-port, while the reflecting material separates shield 2 (stripes) from the bulk material. This buffer area prevents thermal neutrons from scattering in shield 2 back into the bulk material. At the same time, epithermal neutrons escaping from the bulk material are scattered back. This BSA has a larger shield 2, to further decrease beam contamination. Finally, (c) is a modification of (b) with more material aimed at increasing lateral neutron moderation and absorption. To mitigate the unavoidable loss of flux, the volume of reflecting material around the bulk moderator was also increased. The shape of reflector, lateral shielding, and bulk moderator were optimised to reach a satisfactory epithermal neutron flux at the beam-port. This BSA was also intended to better collimate the neutron beam.

Five different beams based on these three geometrical structures were selected changing the materials. Table 1 describes the composition and geometrical set-up of the 5 presented BSAs.

### 2.2. Evaluation of Physical in-Air Parameters

The described beams were chosen among others using the FOM listed in Table 2, representing the physical characteristics recommended by IAEA. Values of the FOMs were obtained by transporting neutrons with MCNP6 [33] from the beryllium target through the BSA to the beam port, where a surface tally with 6 cm radius was computed for the total neutron current (F1 tally type), epithermal neutron flux (F2 tally type), thermal neutron flux, fast neutron, and gamma dose (F2 tally type combined with an FM card with appropriate tissue kerma factors).

### 2.3. Evaluation of the Therapeutic Potential of Beams Using Radiobiological FOMs

To introduce a criterion related to the BNCT treatment outcome, we tested the beams in a phantom representing a human neck bearing a tumor. The phantom is a cylinder (radius of 10 cm and height of 24 cm) of mucosa, which is the tissue at risk in Head and Neck (H&N) BNCT treatments. A 2 cm radius sphere centered 3 cm from the phantom equator surface represents the tumor. Boron concentration was assumed to be the standard in Finland treatments: 15 ppm in blood, which results in 30 ppm in mucosa and 52.5 ppm in tumor [34].

**Figure 2:**
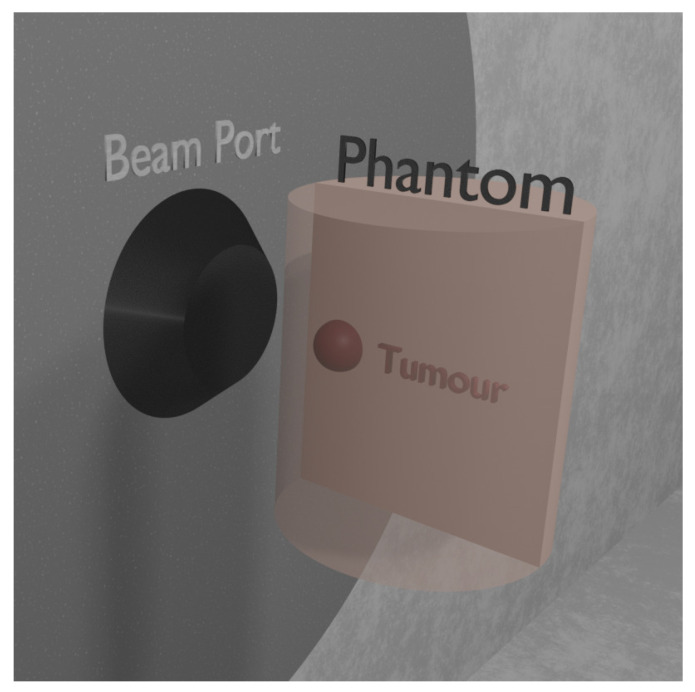
3D view of the phantom simulated in front of the beam port, facing the neutron beam with the tumor region.

Figure 2 shows the simulated set-up, where the phantom is irradiated with a beam-to-phantom distance of 7.5 cm, reproducing the average distance of patients from the beam port in clinical experience [35]. The dose was calculated with the photon iso-effective model [36]. The Uncomplicated Tumor Control Probability (UTCP) [35], defined as the probability to control the tumor without complication in the healthy mucosa, was chosen as the radiobiological FOM. UTCP is the product of the tumor Control Probability (TCP) and the complementary of Normal Tissue Complication Probability (1-NTCP). The closer this FOM is to 1, the higher is the probability to control the tumor without normal tissue complications. This criterion was used for the first time by Provenzano et al. [35] for the comparison of different clinical facilities, and it is proposed here as a criterion to compare the therapeutic potential of different candidate beams. UTCP was computed using the TCP model for inhomogeneous dose distribution [35] and the NTCP model that can predict mucositis of grade 3 or higher (≥G3) after head and neck cancer radiotherapy with photons and BNCT [36]. With this model, it is possible to calculate the treatment time that maximizes UTCP.

### 2.4. Evaluation of the Beams Suitability by Out-of-Beam Dosimetry

When a clinical facility is projected, it is critical to select the safest beam for the patient, i.e., the one delivering the lowest dose to the organs outside the irradiation field. To study this, the beams were tested using the anthropomorphic phantom MIRD [37,38]. An equivalent dose was calculated with the prescription of an ICRP report 116 [39]: weight for photon dose is 1, for the boron component, it is 20, and the neutron dose has been weighted according to the function of energy described in the ICRP report 116. To compare the out-of-beam dose values with those found in literature, a biologically weighted dose was also obtained by multiplying the different components by RBE/CBE shown in Table 3. These doses were computed with MCNP6 coupling neutron fluence with kerma factors.

Boron concentration was assumed to be 15 ppm in all the normal tissues except for the skin and kidneys, set to absorb 22.5 ppm [43] and 75 ppm, respectively. Kidneys are known to uptake a higher boron concentration than other tissues due to the excretion of BPA via the urinary system: pharmacokinetics studies performed in animals and humans using fluorinated BPA and PET imaging have been analyzed [44,45]. Concentration in kidneys is high shortly after the injection of the tracer, and then it decreases after about 1 h to a value around 5 times higher than in other normal tissues. At the same time, the concentration in bladder increases. Bio-distribution studies in a big animal model [43] treated with a protocol equal to the clinical one (350 mg/kg of BPA for 45 min) confirm the findings in the human studies. For this study, we thus assumed a boron concentration in kidneys equal to 75 ppm. As shown in the PET images of the cited papers, a high boron concentration is expected in the urine after some time from BPA administration. However, the urinary bladder tissue absorbs a boron concentration similar to other normal tissues [46]. The dose delivered to the tissues due to the boron present in the urine is limited to the layer of urine adjacent to the organ walls with a thickness equal to the particles range, i.e., about 10 micrometers. For this reason, boron in urine was neglected in the calculation of the dose delivered to the bladder, assumed to uptake 15 ppm of boron.

The combination of therapeutic potential and suitability assessment allowed a clear selection of the best neutron beam design.

#### Treatment Planning Simulation

The selected beam was finally used to simulate a BNCT treatment plan. The chosen clinical case is a Head and Neck cancer patient (squamous cell carcinoma) treated with BPA-mediated BNCT in Finland [34]. The performance of the selected beam is compared with that of FiR 1 in the real treatment.

**Figure 3:**
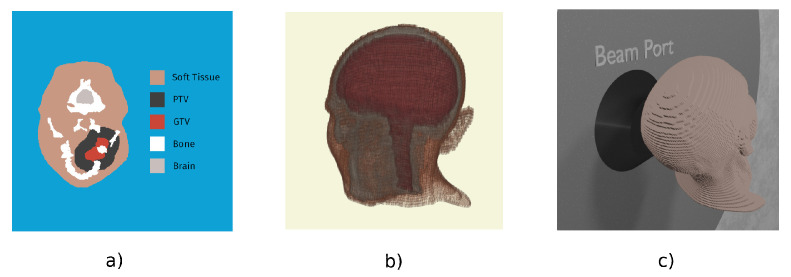
(**a**) shows a slice of the patient DICOM image segmented into five materials, which is then converted into a voxelized phantom by a MultiCell as shown in (**b**); (**c**) shows the TPS set-up as fed into the MCNP simulation.

MultiCell was used as the Treatment Planning System, to obtain the voxelized patient model shown in Figure 3a (segmented image) and in Figure 3b (3D representation), to set the two fractions treatment configuration and to produce the input for dose calculation by MCNP [47,48]. Figure 3c shows the voxelized phantom positioned in front of the beam port. The dose-limiting point in mucosa used to calculate the optimum treatment time and maximum UTCP is the one selected by medical doctors in the real treatment. Based on the obtained irradiation time, photon iso-effective doses and the Dose Volume Histogram (DVH) were calculated in the contoured tumor volume. The UTCP curve was computed as described by Provenzano et al. [35].

## 3. Results and Discussion

### 3.1. Evaluation of Physical in-Air Parameters

Table 4 shows the results of the calculation of physical FOMs for each beam (Table 1 and Figure 1), compared to the recommended values. BSA #1 and BSA #4 ensure a lower gamma contamination while keeping a high neutron flux. This is mainly due to the use of heavy water and graphite, in the shield 2 region, instead of materials containing hydrogen. BSA #2 is the one with the lowest fast neutron dose contamination, while BSA #3 is the one with the lowest gamma dose contamination. These beams show lower fast neutron dose contamination compared to BSA #1 and BSA #4, the main reason being that the shielding material contains hydrogen, which scatters low energy neutrons back into the beam. As a result, the epithermal neutron flux increases, but the epithermal energy spectrum is shifted towards energies lower than 1 keV. BSA #5 is characterized by higher collimation (it is the only one complying with IAEA recommendation for this parameter) at the expense of lower epithermal neutron flux and the highest fast neutron dose contamination.

Table 4 shows how difficult it can be to choose a beam based on fixed values of physical properties. None of the tested beams are clearly optimal compared to the IAEA recommendations. However, they may still be useful for patient treatment. To assess the suitability for clinical use, the beams were tested using the UTCP.

### 3.2. Radiobiological FOM

Table 5 shows the evaluation of the UTCP on the cylindrical phantom, obtained by maximizing this FOM for each BSA configuration. The values of UTCP are very similar for all the beams, with BSA #5 having the highest value, obtained from the highest TCP and lowest NTCP. To understand if these values represent a good indication of therapeutic potential, we can compare these values to the one obtained in the same phantom with the clinical beam Fir 1: 0.45 [35].

It is important to note that UTCP is not used here to establish an absolute medical criterion, but as a FOM to inter-compare beams on the basis of their therapeutic potential. Notably, when the classical physical parameters would not promote any of these beams, UTCP demonstrates that they are all comparable to the reference clinical beam, used for many patients. Thus, with this criterion, they are all potentially useful for treatment. Still, the UTCP alone is not enough to establish a ranking, as all beams give the same UTCP within the uncertainty.

All beams allow reasonable treatment times. Moreover, the maximum dose to the healthy tissue at risk (D) is very close to the clinically prescribed dose of 6 Gy in Finland protocol [49].

### 3.3. Out-of-Beam Dosimetry

**Figure 4:**
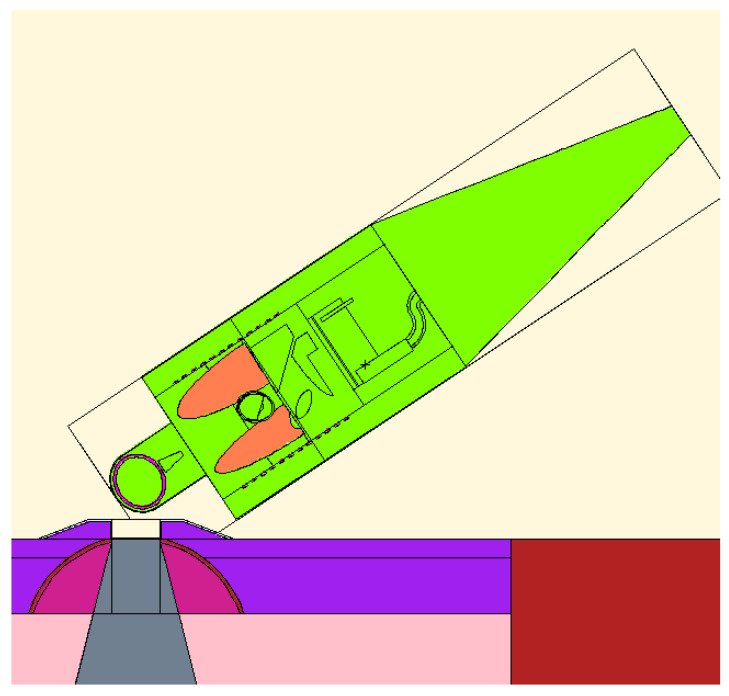
The image shows an MCNP plot of the MIRD phantom position that mimics a typical head irradiation in BNCT.

**Figure 5:**
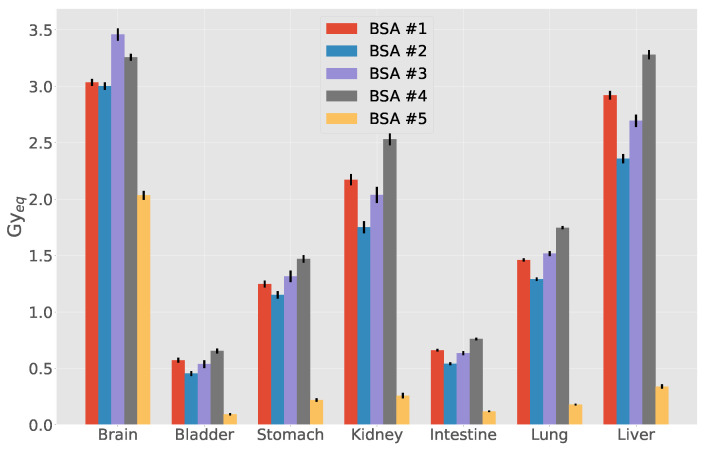
Comparison of out-of-beam dose evaluated in the MIRD phantom. Each BSA is evaluated for its treatment time shown in Table 5.

The anthropomorphic male MIRD phantom was positioned in a representative irradiation position for H&N cancer treatment (Figure 4). For all beams, the biological weighted dose in cGy${}_{Eq}$ has been calculated in all the organs of the phantom. Figure 5 shows the results for all the BSAs, indicating that BSA #5 delivers doses significantly lower than all other configurations to the organs outside the irradiation field. For the particular case of BSA #5, the detailed dosimetry values on the organs of the MIRD phantom are given in Table 6. The values refer to the dose absorbed by a patient in a treatment time as calculated to maximize the UTCP: 24.5 min. The table also reports the absorbed dose (cGy) separating the radiation components, and the weighted dose (cSv), calculated following the prescription of ICRP report 116 [39].

This result together with the therapeutic potential is a clear demonstration that BSA #5 is clinically the most performing beam in terms of therapeutic potential and safety.

#### Comparison with Other Beams

**Figure 6:**
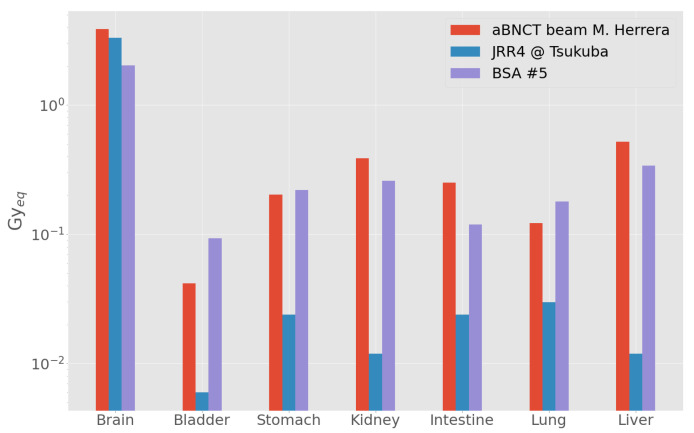
Out-of-beam dose evaluated in the MIRD phantom with BSA #5, with a clinical reactor beam (JRR4) and with the ab-BNCT neutron beam designed by Herrera starting for 2.7 MeV protons coupled with Li target [50].

We compared the results of out-of-beam dosimetry of BSA #5 with those obtained in a similar irradiation position with both a designed ab-BNCT beam and with a reactor beam used in BNCT clinical applications (JRR4, Japan) [29,50]. To this end, the components of absorbed dose were weighted using the RBE/CBE values and considering boron concentration listed in the cited documents. Figure 6 shows that the dose delivered to brain is comparable for the three beams. Furthermore, the dose to peripheral organs delivered by ab-BNCT prototypes are comparable but higher with respect to JRR4 [29].

### 3.4. Characteristics of the Selected Beam

**Figure 7:**
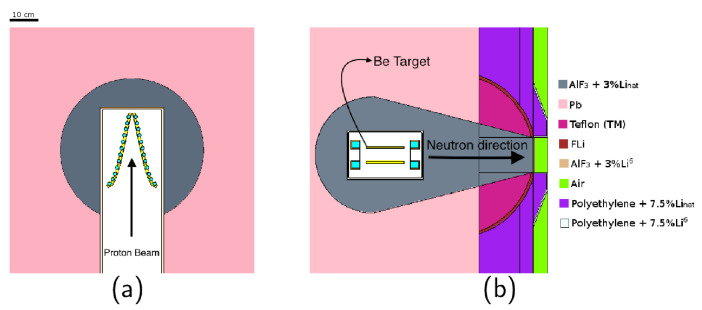
Geometrical configuration of the final BSA, the different colors represent the material composition of the BSA. (**a**) xy plane: the proton beam hits the target in the *z*-direction. (**b**) xz plane. The target has a V-shape, to keep a compact design while removing the total heat generated [15].

**Figure 8:**
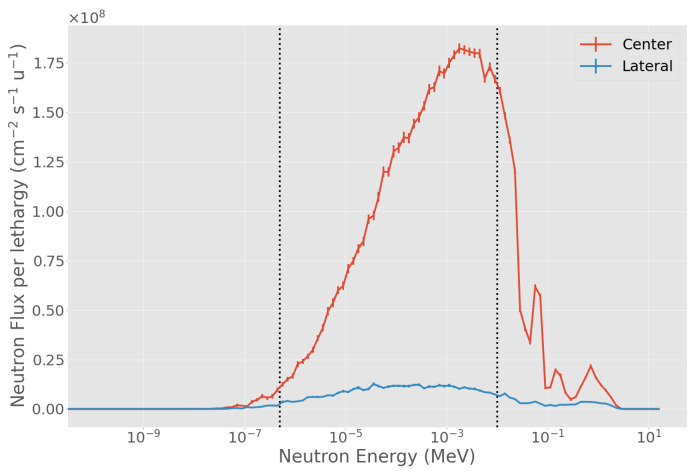
BSA #5 energy spectra at the surface of the beam-port. Blue line: energy spectrum in a 6 cm radius disk, corresponding to the beam-port. Blue line: spectrum in a ring with minimum radius of 6 cm and maximum radius of 12 cm. The two vertical dotted lines separate the thermal-to-epithermal (0.5 eV) and epithermal-to-fast (10 keV) energy ranges.

**Figure 9:**
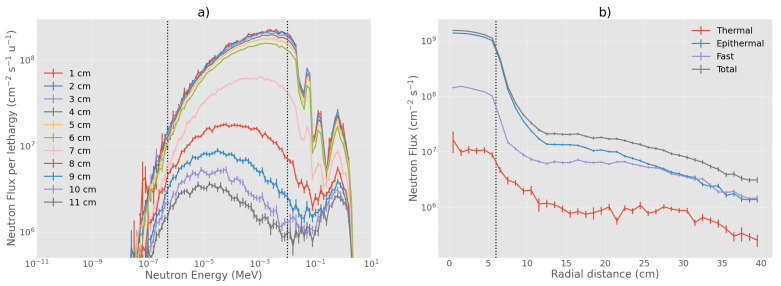
Neutron flux and energy spectra at the beam-port for BSA #5. (**a**) neutron spectra in concentric rings, with radii increasing by 1 cm. The two vertical dotted lines separate the thermal-to-epithermal (0.5 eV) and epithermal-to-fast (10 keV) energy ranges; (**b**) radial neutron flux distribution from the center of the beam-port in concentric rings going from 0 cm to 40 cm of radius. The vertical dotted line delimits the beam-port radius.Blue: thermal neutron flux, orange: epithermal flux, green: fast flux, and red: total neutron flux.

The BSA #5 is showed in detail in Figure 7. As shown in Figure 8, the neutron spectrum averaged over the beam-port area (6 cm radius circle) peaks around the desired energy range between 1 to 10 keV. The flux decreases sharply when averaged over a circular ring going from 6 cm radius to 12 cm radius, the out-of-beam area (Figure 8). Figure 9 shows spectrum and flux behavior in-beam and out-of-beam. In Figure 9a, the logarithmic spectrum averaged over circular sectors of radii between 1 and 11 cm demonstrates that the spectral distribution is maintained below 6 cm (in-beam). Out of beam, the flux decreases with the radius, and the proportion of the spectrum components changes. Figure 9b shows the distribution of the different spectral components as a function of the radius. Flux variation is within 20% inside the beam-port, and it sharply decreases out of beam. The thermal, epithermal, and fast neutron components change at increasing radial distance. This is due to the different material composition of the BSA: on the beam axis, AlF${}_{3}$ moderates the neutron spectra to a peak energy of ≈1 keV while, out of beam, the BSA contains a high concentration of hydrogen and consequently fast and epithermal neutrons are moderated towards thermal energies.

### 3.5. Treatment Planning

The beam from BSA #5 was used to simulate the treatment planning of a real clinical case treated in Finland. The results of the treatment planning simulation for the optimum irradiation time and the corresponding data for FiR 1 are shown in Table 7. For this beam, which only partially satisfied the physical recommendations, the maximum UTCP is comparable to the one obtained with Fir 1, a beam that complies with all the physical FOMs.

**Figure 10:**
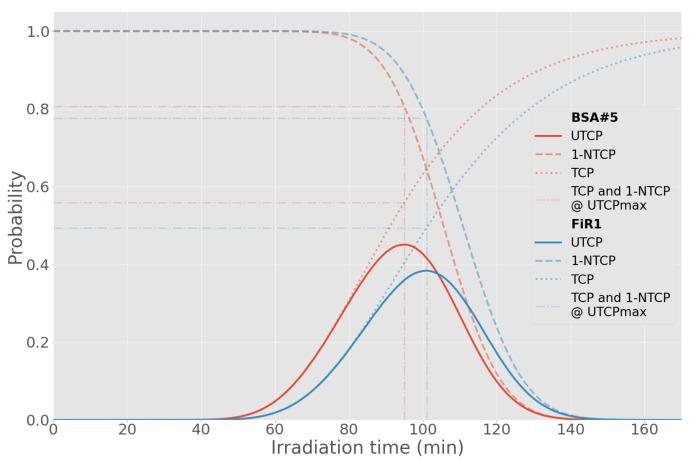
Comparison of the UTCP value for BSA #5 and FiR 1 as a function of the irradiation time.

**Figure 11:**
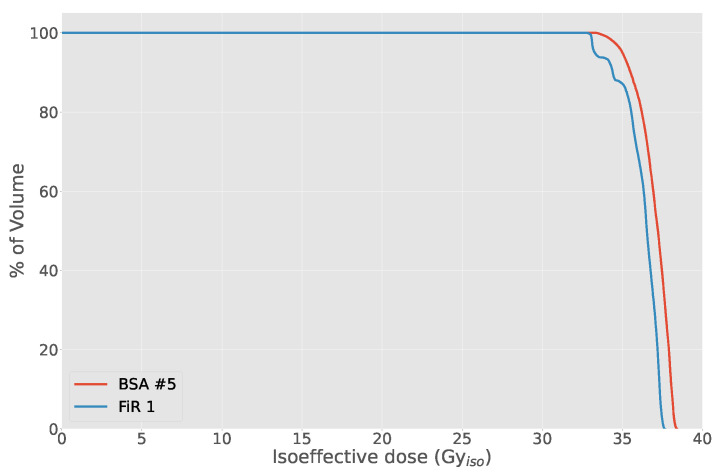
Comparison of the DVH obtained with BSA #5 and FiR 1.

**Figure 12:**
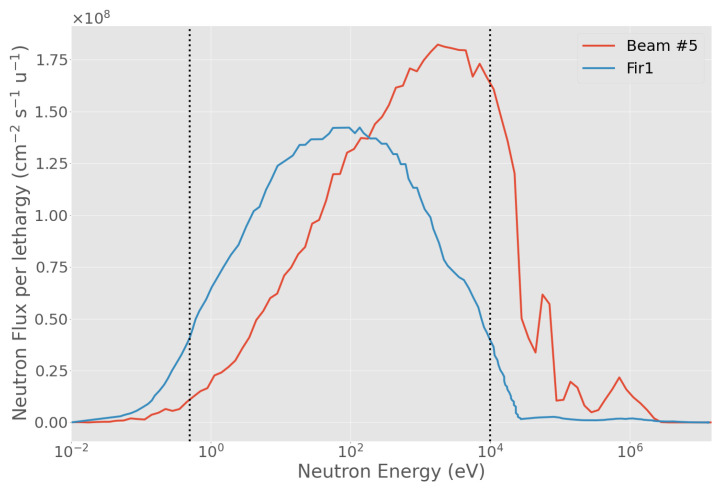
Comparison of the neutron beam spectra for BSA #5 (red line) and FiR 1 [51] (blue line). The two vertical dotted lines separate the thermal-to-epithermal (0.5 eV) and epithermal-to-fast (10 keV) energy ranges.

At the maximum UTCP value for each beam, for approximately the same NTCP (Table 7), the TCP for BSA #5 is 0.56, for FiR 1 is 0.49, as shown in Figure 10. The higher TCP is a consequence of the higher minimum dose delivered to the tumor, as shown in Figure 11. In fact, the beam from BSA #5 has a higher fast component than FiR 1 (Figure 12), thus it is more penetrating.

We used the selected beam to compare its performance with the reference neutron facility FiR 1, in a clinical case that was successfully treated there. If clinical decision is to select the treatment time that maximizes the UTCP, the therapeutic potential of the selected beam—quantified by the most probable value of UTCP—is 15% higher than the one obtained for FiR 1. The real irradiation time of the H&N cancer patient treated with FiR 1 was 110 min, a value slightly higher than the one that maximizes the UTCP. If we now simulate an irradiation time to equate the NTCP of the real treatment, the candidate beam achieves a TCP value 15% higher than the one obtained with FiR 1 in an irradiation time almost identical (i.e., a TCP value of 0.7 in 105 min for BSA #5 VS a TCP value of 0.6 in 110 min for FiR 1).

## 4. Conclusions

This work aimed at designing a clinical BNCT facility based on the accelerator built by INFN, employing innovative methods to select the most performing and the safest candidate among different possibilities. The first step was to choose the BSA generating a beam suitable to treat deep-seated tumors. The free-beam in-air parameters proved to be a descriptive tool and a guide to select possible candidates which however did not allow a fair ranking of the beams. We then introduced the concept of *therapeutic potential*, i.e., the capacity to treat a deep-seated tumor without damaging the tissue at risk. UTCP is a radiobiological FOM which condenses the 3D dose distribution into one value related to the clinical outcome. This FOM demonstrated that beams that did not satisfy the physical recommendations were indeed comparable with a reference clinical beam on the basis of the therapeutic potential. For the selection of a suitable beam, we also evaluated safety, calculating the dose delivered to the other organs of the patient. This figure describes the *suitability* of a BNCT neutron beam. It is not possible to define a threshold above which the dose absorbed in an organ is considered too high for the treatment because the priority is the clinical outcome of the tumor irradiation [39]. Hence, we selected the configuration ensuring the minimum out-of-beam absorbed dose, which was also comparable with another designed accelerator-based neutron beam.

The obtained results confirm that it is possible to produce a neutron beam for clinical BNCT from the INFN RFQ. The chosen beam satisfies only some of the IAEA recommendations. However, it proved to ensure a comparable clinical performance to that of our reference beam FiR 1 for H&N treatment.

Our approach adds to the standard evaluation of the in-air physical parameters, the calculation of suitable radiobiological FOMs and out-of-field dose that directly account for the clinical performance and safety of these beams. The possibility to extend this approach to other tumors, testing the therapeutic potential of the beams for a wider class of treatments, requires the availability of the computational tools, the radiobiological data and the models to calculate the 3D dose distribution and the FOMs to predict the clinical outcome. It is thus particularly important and urgent to produce in-vitro and in-vivo experiments to feed the models. Moreover, suitable phantoms, with simple yet significant geometrical characteristics, and real clinical cases must be selected to allow for a broader evaluation and comparison with existing beams.

An important discussion is now ongoing within the BNCT community on the necessity to establish common guidelines to evaluate BNCT beams. This is a non-trivial issue because the evaluation criteria strictly depend on the type of tumors that are addressed, i.e., shallow or deep-seated, and thus on the spectra that are preferable. Nevertheless, the biological effects of the overall dose distributions in patient must be taken into account. For this reason, radiobiological figures of merit such as TCP, NTCP, and UTCP, give a deeper insight into the clinical effectiveness and outcome of the simulated treatment planning thus providing a robust criterion to predict the beam performance.

## Figures and Tables

**Table 1:** Materials and geometrical configurations of some tested BSA setup. The last column refers to the BSA designs shown in Figure 1.

BSA	Shield 1	Shield 2	Reflector	Bulk Material Composition (Thickness [cm])	Design
#1	Pb	HW + CLi	Pb	AlF${}_{3}$ (35.5) + LiF (1) + Bi (0.5) + Ti (1)	Figure 1b
#2	Pb	C + CH${}_{2}$Li	Pb	AlF${}_{3}$ (35.5) + LiF (1) + Bi (0.5) + Ti (1)	Figure 1a
#3	Pb	C + CLi + CH${}_{2}$Li + LiF	Pb + LiF	AlF${}_{3}$ (35.75) + LiF (1.5) + Bi (0.5) + Ti (1)	Figure 1b
#4	Pb	C + CLi	Pb	AlF${}_{3}$ (35.5) + LiF (1) + Bi (0.5) + Ti (1)	Figure 1b
#5	Pb	LiF + Teflon	CH${}_{2}$Li	AlF${}_{3}$ with 3% mass of LiF (37)	Figure 1c

**Table 2:** IAEA recommended FOM [27].

$\phi_{\mathrm{epi}}$	$\frac{\phi_{\mathrm{th}}}{\phi_{\mathrm{epi}}}$	$\frac{{\dot{D}}_{\mathrm{Fast}}}{\phi_{\mathrm{epi}}}$	$\frac{{\dot{D}}_{\gamma }}{\phi_{\mathrm{epi}}}$	$\frac{J}{\phi_{\mathrm{epi}}}$
10${}^{9}$ (cm${}^{-2}$ s${}^{-1}$)	-	10${}^{-13}$ (cm${}^{2}$ Gy)	10${}^{-13}$ (cm${}^{2}$ Gy)	-
>1	<0.05	<2.0	<2.0	>0.7

**Table 3:** RBE and CBE factors employed to calculate a biological dose in different tissues [40,41,42].

Tissue	RBE${}_{\gamma }$	RBE${}_{n}$	CBE${}_{B}$	${}^{10}$B Concentration (μg/g)
Brain	1	3.2	1.3	15
Skin	1	2.5	2.5	22.5
Liver	1	3.2	4.2	15
Lung	1	3.2	1.3	15
Kidney	1	3.2	1.3	75
Bladder	1	3.2	1.3	15
Tumor	1	2.2	5.3	52.5

**Table 4:** IAEA FOM calculated for the BSAs listed in Table 1. The reported results have a relative error lower than 2%.

	$\phi_{\mathrm{epi}}$	$\frac{\phi_{\mathrm{th}}}{\phi_{\mathrm{epi}}}$	$\frac{{\dot{D}}_{\mathrm{Fast}}}{\phi_{\mathrm{epi}}}$	$\frac{{\dot{D}}_{\gamma }}{\phi_{\mathrm{epi}}}$	$\frac{J}{\phi_{\mathrm{epi}}}$
	10${}^{9}$ (cm${}^{-2}$ s${}^{-1}$)	-	10${}^{-13}$ (cm${}^{2}$ Gy)	10${}^{-13}$ (cm${}^{2}$ Gy)	-
**Recommended**	**>1**	**<0.05**	**<2.0**	**<2.0**	**>0.7**
BSA #1	2.58	0.019	9.16	3.96	0.59
BSA #2	2.56	0.054	6.70	6.66	0.60
BSA #3	1.73	0.004	7.71	2.70	0.62
BSA #4	2.79	0.018	9.09	3.78	0.59
BSA #5	1.08	0.009	9.50	4.17	0.74

**Table 5:** Results of the simulations with the cylindrical phantom. Each UTCP value is followed by its TCP, NTCP, and irradiation time T${}_{irr}$. The uncertainty corresponds to one standard deviation. For the UTCP, this corresponds to the standard deviation of the product of the independent random variables TCP and (1-NTCP). The dose column D shows the maximum dose to the healthy tissue, while the % Dose section displays the different contributions to the dose D. The last row lists the simulations’ results for the reference beam Fir 1 [35].

BSA	T${}_{\mathrm{irr}}$ (min)	UTCP	TCP	NTCP	D (Gy)	% Dose			
						(n,α)	(n,p)	(n,n’)	γ
#1	10	0.41 ± 0.06	0.50 ± 0.04	0.19 ± 0.11	5.79	61	6	6	27
#2	12.5	0.40 ± 0.07	0.52 ± 0.04	0.23 ± 0.13	6.15	59	5	6	30
#3	20	0.44 ± 0.07	0.57 ± 0.05	0.23 ± 0.10	5.81	62	7	6	25
#4	9.5	0.40 ± 0.07	0.54 ± 0.05	0.26 ± 0.11	5.98	61	6	6	27
#5	24.5	0.47 ± 0.07	0.58 ± 0.05	0.18 ± 0.09	5.59	62	9	6	23
Fir 1	33	0.45 ± 0.07	0.54 ± 0.05	0.17 ± 0.08	4.80	78	2	8	12

**Table 6:** Absorbed dose (cGy), equivalent dose (cSv) and biologically weighted dose (cGy_Eq) to the organs of a MIRD phantom for BSA #5. Dose is evaluated considering the whole treatment time of 25.4 min.

Organ	D${}_{N}$ (cGy)	D${}_{B}$ (cGy)	D${}_{H}$ (cGy)	D${}_{\gamma }$ (cGy)	D${}_{W}$ (cSv)	D${}_{\mathrm{Eq}}$ (cGy_Eq)
Brain	1.060	1.210	41.4	43.8	511.0	203 ± 4
Bladder	0.106	0.122	0.1	6.3	48.0	9 ± 1
Stomach	0.151	0.174	1.0	14.9	77.0	22 ± 2
Kidneys	0.121	0.139	0.3	11.0	226.0	26 ± 2
Intestine	0.105	0.120	0.3	8.3	51.0	12 ± 1
Lungs	0.188	0.216	2.9	0.6	80.0	18 ± 1
Liver	0.133	0.153	0.8	11.5	112.0	34 ± 2
Heart	0.187	0.215	2.2	16.6	95.0	28 ± 2
Spleen	0.121	0.140	1.0	14.5	65.0	21 ± 2
Head	0.551	0.631	19.9	37.2	282.0	115 ± 2
Thyroid	0.483	0.554	9.1	26.8	232.0	68 ± 7
Testicles	0.078	0.090	0.1	7.1	38.0	9 ± 1
Pancreas	0.107	0.124	0.3	9.1	53.0	13 ± 1
Pharynx	0.712	0.818	14.5	33.6	337.0	98 ± 8
Marrow	0.145	0.166	4.9	16.0	80.0	35 ± 1
Adrenal	0.091	0.105	1.0	10.1	48.0	16 ± 4
Thymus	0.249	0.287	1.6	17.7	120.0	29 ± 4
Skin	0.173	0.198	6.8	20.6	128.0	54 ± 1
Trunk	0.143	0.165	1.8	14.6	74.0	24 ± 1

**Table 7:** Results of the treatment planning simulation using BSA #5 and corresponding values for FiR 1. The maximum UTCP, TCP, and NTCP are reported for the optimum irradiation time T_*irr*_ of each beam. T_*irr*_ corresponds with the maximum UTCP value, where the probability of controlling the tumor of the patient without normal tissue complication is maximized [35]. Dose D is the maximum absorbed dose to the dose-limiting point in healthy tissue, and the % Dose displays the different contributions to the dose D.

Beam	T${}_{\mathrm{irr}}$ (min)	UTCP	TCP	NTCP	D (Gy)	% Dose		
						(n,α)	(n,p) and (n,n’)	γ
BSA #5	95	0.45 ± 0.07	0.56 ± 0.05	0.19 ± 0.11	6.0	62	8	30
FiR 1	101	0.38 ± 0.07	0.49 ± 0.05	0.22 ± 0.12	5.4	74	6	20

## Data Availability

The BSA models, neutron source, and phantoms presented in this study are available upon request from the authors. To compute the dosimetric model and evaluate the UTCP, refer to [36,52]; to perform the calculation, use https://bnct.com.ar/calculator.html (accessed on 10 December 2020). The photon iso-effective dose calculator is available at https://bnct.com.ar/ (accessed on 10 December 2020).

## Abbreviations

The following abbreviations are used in this manuscript:

BNCT	Boron Neutron Capture Therapy
BSA	Beam Shaping Assembly
INFN	Istituto Nazionale di Fisica Nucleare
IAEA	International Atomic Energy Agency
TCP	Tumor Control Probability
NTCP	Normal Tissue Complication Probability
UTCP	Uncomplicated Tumor Control Probability
FOM	Figure of Merit
RFQ	Radio Frequency Quadrupole
H&N	Head and Neck
